# Temporal patterns of damage and decay kinetics of DNA retrieved from plant herbarium specimens

**DOI:** 10.1098/rsos.160239

**Published:** 2016-06-22

**Authors:** Clemens L. Weiß, Verena J. Schuenemann, Jane Devos, Gautam Shirsekar, Ella Reiter, Billie A. Gould, John R. Stinchcombe, Johannes Krause, Hernán A. Burbano

**Affiliations:** 1 Research Group for Ancient Genomics and Evolution, Department of Molecular Biology, Max Planck Institute for Developmental Biology, Tuebingen 72076, Germany; 2 Department of Molecular Biology, Max Planck Institute for Developmental Biology, Tuebingen 72076, Germany; 3 Institute of Archaeological Sciences, University of Tübingen, Tuebingen 72076, Germany; 4 Departments of Paleoanthropology and Archaeogenetics, Senckenberg Center for Human Evolution and Paleoenvironment, University of Tübingen, Tuebingen 72076, Germany; 5 Department of Ecology and Evolutionary Biology, Toronto, Ontario, CanadaM5S; 6 University of Toronto, Toronto, Ontario, CanadaM5S; 7 Max Planck Institute for the Science of Human History, Jena 07743, Germany

**Keywords:** ancient DNA, DNA damage, herbarium, DNA decay

## Abstract

Herbaria archive a record of changes of worldwide plant biodiversity harbouring millions of specimens that contain DNA suitable for genome sequencing. To profit from this resource, it is fundamental to understand in detail the process of DNA degradation in herbarium specimens. We investigated patterns of DNA fragmentation and nucleotide misincorporation by analysing 86 herbarium samples spanning the last 300 years using Illumina shotgun sequencing. We found an exponential decay relationship between DNA fragmentation and time, and estimated a per nucleotide fragmentation rate of 1.66 × 10^−4^ per year, which is six times faster than the rate estimated for ancient bones. Additionally, we found that strand breaks occur specially before purines, and that depurination-driven DNA breakage occurs constantly through time and can to a great extent explain decreasing fragment length over time. Similar to what has been found analysing ancient DNA from bones, we found a strong correlation between the deamination-driven accumulation of cytosine to thymine substitutions and time, which reinforces the importance of substitution patterns to authenticate the ancient/historical nature of DNA fragments. Accurate estimations of DNA degradation through time will allow informed decisions about laboratory and computational procedures to take advantage of the vast collection of worldwide herbarium specimens.

## 1. Introduction

Under favourable conditions DNA fragments can survive in plant [1] and animal tissues [2] for hundreds of thousands of years providing a molecular record of the past. Therefore, the examination of historical genomes permits the inclusion of temporal data into evolutionary studies, which allows a more accurate inference of rates and timing of key evolutionary events. The vast majority of ancient DNA (aDNA) studies have focused on animal remains, whereas plant remains have received less attention [3] despite the abundance of historic plant specimens.

DNA retrieved from historic specimens comes in small amounts and is a mixture of endogenous and microbial DNA that either was present pre-mortem or colonized the tissue post-mortem [4]. The aDNA comes in small fragment sizes [5] and holds various modifications that distinguish it from DNA extracted from fresh tissue [6]. DNA fragmentation is partially driven by spontaneous depurination and subsequent hydrolysis of the DNA backbone [7,8]. The typical sign of depurination, which is the excess of both adenine (A) and guanine (G) before DNA breaking points, has been detected by high throughput sequencing (HTS) in libraries constructed from aDNA [9]. DNA degradation is additionally marked by an increase of cytosine (C) to thymine (T) substitutions towards the end of aDNA fragments. This pattern results from spontaneous deamination of C residues to uracils (U) that are read as T by the polymerase and occur in higher proportion in single-stranded DNA overhangs [9,10]. A biochemical definition of aDNA includes all above-mentioned characteristics but does not delineate a time boundary between ancient and modern DNA [3].

It is interesting to understand quantitatively how these aDNA-associated patterns change through time, as they could be used to both authenticate DNA fragments retrieved from historic samples of different ages, and to calculate DNA decay rate based on their fragmentation patterns [2]. Using animal remains it has been found that there is a strong positive correlation between the amount of putative deamination (excess of C to T substitutions) and the sample age [11]. Hence, the excess of C to T substitutions has been used as a criterion of authenticity in aDNA studies [12]. The correlation between other aDNA-associated patterns and sample age is weaker [11], which could be a consequence of the different environmental conditions in which fossils were preserved, processed and stored (e.g. freshly excavated ancient bones are best for amplification of aDNA [13]). To reduce the effect of environmental variation on DNA degradation, a more spatially constrained sample of animal remains has been studied [2]. Allentoft *et al*. [2] calculated the long-term DNA decay rate in bone tissue, which could be used to estimate DNA half-life and, consequently, to put a boundary on how far back in the past DNA could be theoretically retrieved. Because most of the bone samples in Allentoft *et al*. [2] were analysed only by quantitative PCR and not by HTS, it was not possible to investigate how the signals left by deamination or depurination correlate with time in a spatially constrained sample.

Since herbaria contain time snapshots of global biodiversity and could be informative to address a broad spectrum of biological questions, it is fundamental to understand how DNA survives in this type of specimens. Investigating the effect of time on DNA degradation is normally difficult, since environmental conditions such as temperature, pH and humidity, among others, affect DNA stability [14]. Therefore, it is highly advantageous that herbaria samples are prepared and stored using standardized procedures, which reduces the effect of environmental variation among herbaria samples compared with ancient bones. Consequently, herbarium samples are ideal to study the temporal patterns of damage and decay kinetics of DNA. In this study, we analysed 86 herbarium samples collected over the last 300 years using library-based methods coupled with HTS, and produce for the first time an in-depth description of aDNA-associated patterns and its dynamics through time. Additionally, we use the power of multiple DNA sequencing libraries to calculate DNA decay rate in plant desiccated tissue.

## 2. Results

### 2.1. DNA fragmentation

We used a group of multiple species herbarium samples and also freshly prepared (less than 1 year old) herbarium samples of *Arabidopsis thaliana* dried using a wooden press (table 1; electronic supplementary material, table S1). From here on, we will refer to these groups as historic and modern herbarium, respectively. A fraction of historic herbarium samples from Solanaceae have lesions compatible with *Phytophthora infestans* infection and have been previously studied [15]. Since we expect that DNA retrieved from historic samples will be highly fragmented, it is likely that a fraction of the molecule will be covered by both the forward and reverse read. After adapter trimming forward and reverse reads were merged, requiring an overlap of 10 base pairs (bp) between them. We were able to merge on average 96% (83–99%) of the reads from historic herbarium samples, whereas on average 21% (18–40%) of reads could be merged from modern herbarium samples, due to the presence of much longer DNA fragments (electronic supplementary material, table S2). In the modern herbarium samples, the mean of the fragment length distribution corresponded to the fragment size intended during sonication (400 bp) and the merged reads were located at the left tail of the fragment length distribution (electronic supplementary material, figure S1). For all further analysis correlating DNA fragmentation with time we used only merged reads from historic herbarium samples.

**Table 1.** Type and number of herbarium samples.

type of sample	species	number of samples	collection year (range)	number of infected samples^a^
historic	*Arabidopsis thaliana*	54	1863–1993	—
	*Solanum tuberosum*	12	1845–1896	12
	*Solanum lycopersicum*	5	1737–1876	2
	total	71	1737–1993	14
modern	*Arabidopsis thaliana*	15	2014	—

^a^Samples with lesions are compatible with *Phytophthora infestans* lesions.

**Figure 1.**
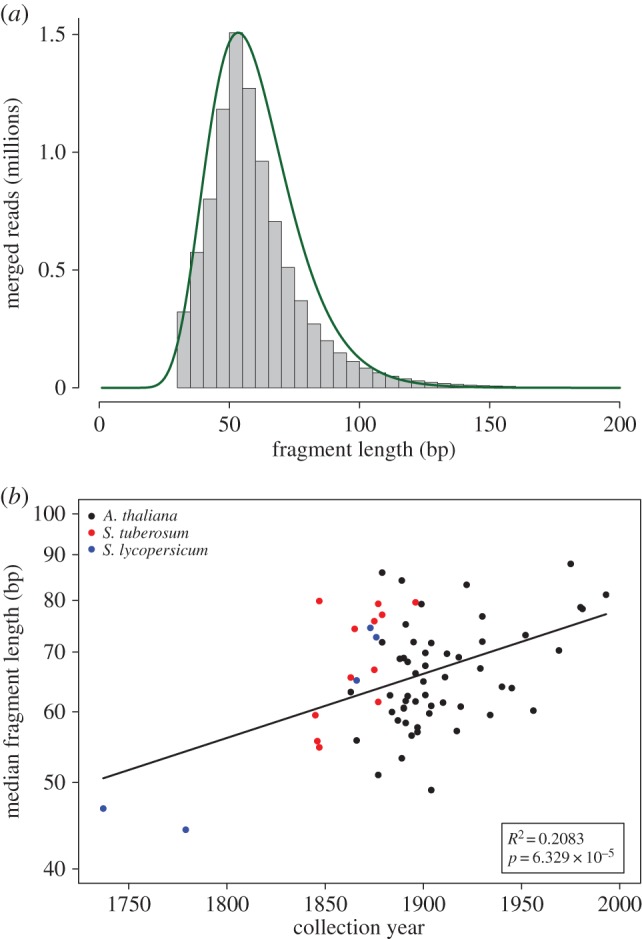
DNA fragmentation. (*a*) Distribution of fragment lengths of merged reads from *A. thaliana* sample NY1365354. The green line shows the fit between the empirical and the lognormal distribution. (*b*) Median length of merged reads as a function of collection year (*N* = 71). The line indicates the linear regression. The inset shows the regression statistics between the natural logarithm of median length and collection year. The *y*-axis is log-scaled and shows, therefore, that the correlation is exponential.

The distribution of fragment lengths of merged reads is not normally distributed and could be better described by a lognormal distribution (figure 1*a*). To evaluate the correlation between fragment lengths with the collection year of each sample, we chose the log-mean value of a fitted lognormal distribution. The regression between the log-mean fragment length and the sample collection year was statistically significant (*R*^2 ^= 0.2; *p* = 6.33 × 10^−5^; *N* = 71; figure 1*b*). To check if the signal was driven only by the oldest eighteenth century *Solanum lycopersicum* samples (figure 1*b*), we repeated the analysis only for the *A. thaliana* samples and found that the regression was still significant (*R*^2^ = 0.175; *p* = 1.6 × 10^−3^; *N* = 54), which implies that the signal arises from the whole set of herbarium specimens and is not driven only by the oldest samples. Since DNA was extracted from some herbarium specimens using CTAB (cetyl-trimethyl ammonium bromide) and PTB (*N*-phenacylthiazolium bromide) extraction protocols [16], we evaluate the effect of these methods on the length distribution of DNA reads and found no difference between them (*p* = 0.75; *N* = 54; electronic supplementary material, figure S2).

### 2.2. DNA break points

It is possible using reads mapped to their respective reference genome to analyse the genomic nucleotide context surrounding the ends of the DNA fragments, and thus look indirectly at DNA break points. We found an excess of purine frequency (both adenine and guanine) in DNA retrieved from historic herbarium samples at position −1 (5′ end; electronic supplementary material, figure S3*a*). This pattern was not found in modern herbarium samples (electronic supplementary material, figure S4*a*), hence in all further analysis correlating DNA breaking points and time we used only historic herbarium samples. We calculated the relative enrichment in purine frequency of both adenine and guanine at position −1 compared with position −5. We then correlate these signatures of depurination with the collection year of the sample. Neither adenine (electronic supplementary material, figure S3*b*) nor guanine (electronic supplementary material, figure S3*c*) relative enrichment showed a significant correlation with collection year. Additionally, we did not find a difference between the average relative enrichment of adenine when compared with guanine (electronic supplementary material, figure S3*b*,*c*). When we analyse independently chloroplast-derived reads, we found purine enrichment at position −1, and no correlation between the relative enrichment of purines and collection year. There were no significant differences between nuclear- and chloroplast-derived reads (*p*(adenine) = 0.34; *p*(guanine) = 0.7; *N* = 71; electronic supplementary material, figure S5).

### 2.3. DNA decay rate

**Figure 2.**
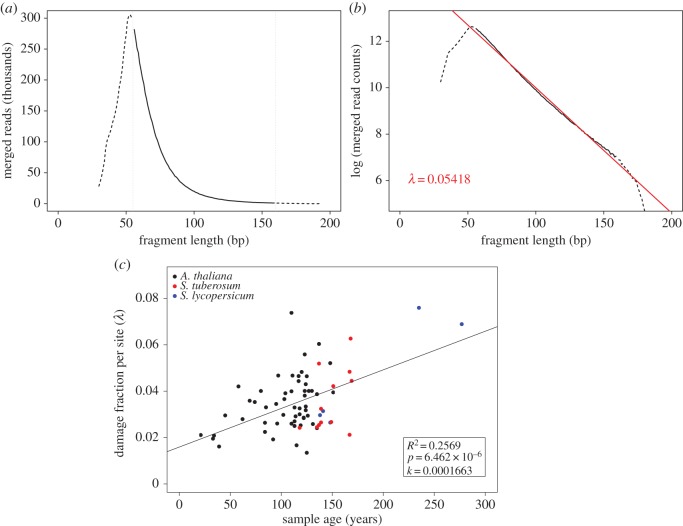
DNA fragmentation and decay rate. (*a*) Distribution of fragment lengths of merged reads from *A. thaliana* sample NY1365354. The solid line, which is surrounded by horizontal dotted lines, shows the part of the distribution that follows an exponential decline. (*b*) Distribution of fragments length for the same library using a *y*-axis with a logarithmic scale. The slope of the exponential part of the distribution (red line) corresponds to the damage fraction per site (*λ*). (*c*) Damage fraction per site (*λ*) as a function of sample age (*N* = 71). The slope of the regression corresponds to the DNA decay rate (*k*) following the formula: *λ *= *k * × age.

The length distribution in aDNA libraries shows an exponential decline as the result of random fragmentation (figure 2*a*) [17]. After logarithmic transformation of the fragment length frequencies, the exponential decline can be described by a linear function with slope *λ*, which corresponds to the damage fraction per site (figure 2*b*). Damage should be interpreted here as DNA bond breaking. To get the overall decay rate for all herbarium samples, we analysed the relationship between *λ* and the age of each sample and found a linear relation. The slope corresponds to the overall per nucleotide decay rate *k* = 1.66 × 10^−4^ per year (*R*^2^ = 0.26; *p* = 6.46 × 10^−6^; *N* = 71; figure 2*c*), which turned out to be six times faster than the rate estimated based on ancient bones, *k* = 2.71 × 10^−5^ per site per year [2].

### 2.4. Nucleotide misincorporation

**Figure 3.**
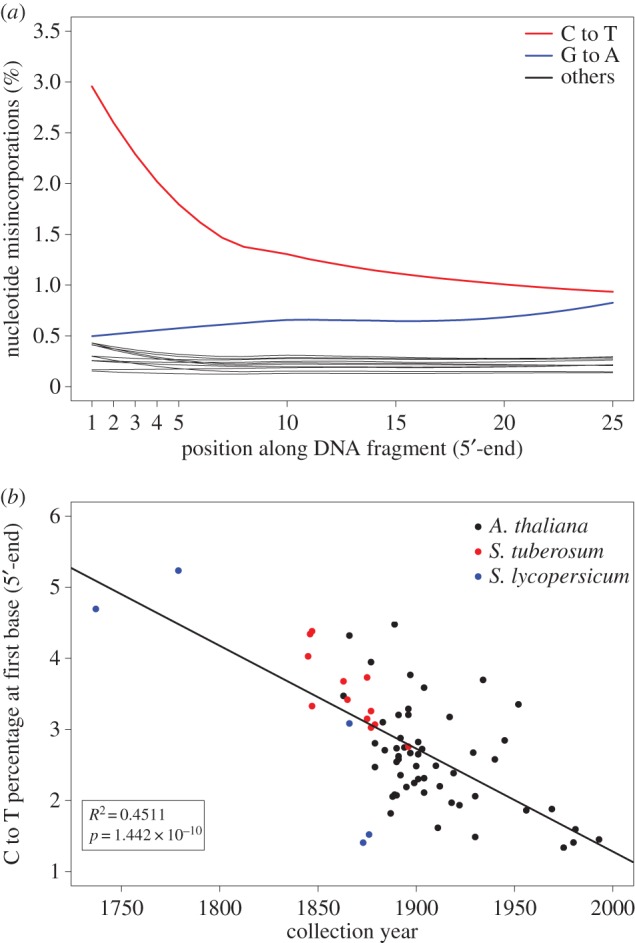
Nucleotide misincorporation. (*a*) Nucleotide misincorporation profile at 5′-end of the reads of *A. thaliana* sample NY1365354. The red line shows an excess of C to T substitutions at the beginning of the read that declines exponentially inwards. (*b*) C to T percentage at first base (5′-end) as a function of collection year (*N* = 71). The C to T percentage and the collection year have a linear relationship.

The most abundant miscoding lesions in aDNA are C to T substitutions, which is caused by deamination of C to U. The U is then read as T by the polymerase during sequencing [9]. The excess of C to T substitutions occurs primarily at the ends of the reads and declines exponentially inwards. We found this pattern present in all historic herbarium samples analysed (figure 3*a*), but absent in modern herbarium samples (electronic supplementary material, figure S4*b*). For all further analysis correlating nucleotide misincorporation with time, we used only historic herbarium samples. Since the excess of C to T substitutions is more manifest at first base, we chose, as previously described [11], the percentage of C to T substitutions at first base as a proxy for miscoding lesions and correlate this value with the samples' collection year (figure 3*b*). We found a very strong linear relationship between these two values (*R*^2^ = 0.45; *p* = 1.44 × 10^−10^; *N* = 71). As it was previously carried out in the DNA fragmentation part, we sought to investigate the effect of the oldest eighteenth century *S. lycopersicum* samples in the correlation between deamination and time. Therefore, we repeated the analysis using only the *A. thaliana* samples and found that the regression was weaker but still significant (*R*^2^ = 0.27; *p* = 5.5 × 10^−5^; *N* = 54). Again, we conclude that the signal arises from the whole set of samples and is not driven only by the oldest samples.

For the infected samples, we also calculated the percentage of C to T substitutions in *P. infestans* derived reads at first base and found the same signature, although it was weaker than the signal found in their host plant (electronic supplementary material, figure S6).

### 2.5. Differences between nuclear- and chloroplast-derived reads

We found that chloroplast-derived reads showed a slightly lower decay rate than the nuclear-derived reads (*k*_chloroplast _= 1.29 × 10^−4^; electronic supplementary material, figure S7*a*). To test if the two decay rates were different, we performed an ANOVA that showed significant effects of both sample age and origin of DNA (nuclear- or chloroplast-derived) on the rate of bond breaking (*λ*) (*Pr*(sample age) = 4.84 × 10^−8^, *Pr*(DNA origin) = 0.012; *N* = 71). However, the effect of DNA origin was very small and there was no significant interaction between sample age and DNA origin (*Pr*(Sample age : DNA origin) = 0.46; *N* = 71). This indicates that the slopes of the two regressions, which correspond to the decay rate *k*, do not differ significantly.

The chloroplast-derived reads show a lower excess of C to T substitutions than the nuclear-derived reads (electronic supplementary material, figure S7*b*). The ANOVA showed in this case highly significant effects of sample age and DNA origin on the percentage of deamination at first base (*Pr*(sample age) = 1.78 × 10^−14^, *Pr*(DNA origin) = 5.69 × 10^−9^; *N* = 71). However, there was no significant interaction between sample age and DNA origin (*Pr*(sample age : DNA origin) = 0.075; *N* = 71). This indicates that nuclear- and chloroplast-derived sequences differ significantly in the extent of deamination (the intersect of the regressions), but not in its rate (slope of the regressions).

## 3. Discussion

Herbaria contain millions of dried plant specimens that provide a record of worldwide changes in biodiversity spanning five centuries. Although plants were not originally collected and stored for genetic studies, the value of these collections as source of DNA has been long recognized by plant biologists [18]. There are a larger number of studies that have used PCR-based approaches to survey these collections, but only a handful of endeavours have used library-based methods coupled with HTS [15,19,20]. Since herbaria collections are an invaluable source of genetic information, it is important to investigate in detail both the properties of DNA retrieved from them and the rate at which DNA damage takes place through time.

### 3.1. DNA fragmentation and decay rate

We confirmed the highly fragmented nature of DNA retrieved from herbarium samples [15,20] (figure 1*a*). The DNA fragmentation is comparable with the level found in animal remains that are several hundreds or even thousands of years old [11], although our samples are utmost 278 years old. In contrast with animal remains [11], we found a weak but significant exponential relation between fragment length and collection year, where more recent samples have longer DNA fragments (figure 1*b*). The lower levels of environmental variation experienced by herbarium samples relative to animal remains could have increased the signal-to-noise ratio allowing the detection of the relation between time and DNA fragmentation.

Since depurination can be inferred by examining DNA breaking points in HTS data [9,11], we analysed our sequencing libraries for an excess of purines at genomic positions surrounding sequencing reads. Both A and G were found overrepresented upstream of the 5′-end break points (electronic supplementary material, figure S3*a*), but no correlation was found between the relative fold enrichment of either A or G and collection year (electronic supplementary material, figure S3*b*,*c*), which implies that the contribution of depurination to DNA breakage does not change through time.

DNA decay and degradation can be understood as a two-step process, with a first rapid phase where the damage is caused mainly by nucleases and digestion by microorganisms, and a second phase where the damage is driven by hydrolytic and oxidative reactions that occur at a much lower rate than the first phase [21]. The correlation between fragmentation and time might be the result of a process occurring in the second phase that can be only detected in samples that have experienced very similar environments, as is the case for herbarium samples.

Modern herbarium samples did not show any age-related fragmentation or excess of purines at DNA breaking points. In fact, the distribution of fragment lengths was centred on the intended fragment length during sonication (electronic supplementary material, figure S1). It has been suggested based on PCR-based methods that most DNA fragmentation in herbarium samples occurs during sample desiccation (drying at 60°C for 18 h) before they are fixed on herbarium sheets, and only a small portion of damage could be attributed to long-term storage [22]. We did not find the sample preparation effect in our herbarium samples however, on the contrary to previous studies [22], we did not use heating to dry our herbarium samples, as it is well established that heat increases the rate of depurination and subsequently β elimination leading to DNA strand breaks [8].

We found that the DNA decay rate in herbarium samples is about six times faster than the rate in bones [2]. It is possible that this big difference could be explained by the characteristic nature of each tissue. In bone, DNA is adsorbed to hydroxyapatite, which decreases the rate of depurination compared with free DNA [14]. Additionally hydroxyapatite binds nucleases [23], which further prevents DNA degradation, especially in the first rapid phase of DNA degradation. DNA in plants' desiccated tissue might be less protected and more exposed to enzymatic and chemical damages. Furthermore, the vast majority of herbarium samples are not mounted on acid-free paper. Acidic paper was regularly used, which could have contributed to DNA degradation, as acid pH increases the rate of depurination *in vitro* [8]. We calculated independently nuclear and chloroplast DNA decay rates and found that the chloroplast DNA decay rate is 0.75 times the nuclear rate (electronic supplementary material, figure S7*a*). In ancient bone, the mitochondrial DNA decay rate is 2–2.5 times slower than the nuclear one [2], in agreement with a study that reported a better preservation of mitochondrial relative to nuclear DNA in permafrost mammoth remains [24]. The slower decay rate in organelle DNA might be a consequence of its circular structure, which makes DNA less accessible to endonucleases [2]. An early report of equal rates of degradation between nuclear and chloroplast DNA in herbarium samples was based on a smaller dataset only interrogated by PCR-based methods, and could be a consequence of lacking experimental resolution [22].

### 3.2. DNA misincorporation

We observed an increase in the percentage of C to T substitutions at the end of the molecule (figure 3*a*) and found a strong correlation between deamination and age (figure 3*b*), as has been found using animal remains [11]. Although chloroplast reads were less deaminated, the correlation between deamination and age held also for them (electronic supplementary material, figure S7*b*). Notably, modern herbarium samples did not show excess of any misincorporation and resembled DNA extracted from fresh tissue.

Since the signal of C deamination has been found recurrently in aDNA studies and there is a strong positive relationship between deamination and sample age [11], the presence of deamination patterns in aDNA HTS studies has been proposed as an authenticity criterion [12]. It is remarkable that C to T substitutions from both animal remains [11] and our data correlate strongly with time, although at a different rate in the two tissues, which implies that deamination is strongly related to the phase two of slow DNA degradation. An excess of C to T substitution at the end of the molecule has been also found in plant [15] and human pathogens [25–27] DNA. We found here that the deamination in plant-pathogen-derived reads is intermediate between nuclear- and chloroplast-derived reads (electronic supplementary material, figure S6). However, we think that the signal is sufficient to be used as an authenticity criterion. We do not suggest that a sample of given age should match a given level of deamination, but instead propose that the excess of C to T at the DNA ends, independent of its magnitude, should be presented as evidence for authenticity. In the future—given an appropriate depth of coverage—it might be possible to also use deamination patterns to authenticate metagenomic aDNA derived from plant or animal tissue, or from environmental DNA profiling.

### 3.3. Practical implications

On the contrary to historic herbarium samples, modern herbarium samples resembled DNA extracted from fresh tissue, which shows that drying by pressing is an ideal method to collect plant samples in long field trips. This also implies that the magnitude of damage that happens in the first phase is highly dependent on the method used to prepare the herbarium specimen.

DNA misincorporations can be confused with natural variation, which will compromise variant calling and increase terminal branches in a phylogenetic context. Both effects are especially prominent in highly deaminated (old) samples that are sequenced at low coverage. Fortunately, it is now possible to almost eliminate this source of error either by removing uracils from DNA molecules during library preparation [28] or by statistically distinguishing true variants from aDNA-associated misincorporations post-sequencing, in reads derived from single-stranded library preparation methods [29].

Since DNA in dried tissue degrades rapidly, the retrieval of DNA from very old samples will require the use of DNA and library extraction preparation methods capable of recovering short length molecules [29,30]. The high DNA fragmentation of historic herbarium samples poses a challenge to genome reduced-representation methods such as RAD (restriction site associated markers)-sequencing [31,32], which has shown low DNA yields and low percentage of reads that could be mapped to the reference genome [33]. Thanks to improvements in library preparation and HTS accuracy, it is possible to sequence and perform mapping-guided assemblies of complete genomes from historic specimens with quality that matches genomes derived from modern specimens and, therefore, exploit the millions of plant remains stored in herbaria worldwide.

## 4. Material and methods

### 4.1. Previously published datasets

Sequences derived from *Solanum tuberosum* and *Solanum lycopersicum* infected by *P. infestans* are deposited in the European Nucleotide Archive, with accession number PRJEB1877.

### 4.2. New datasets

New DNA sequences are deposited in the European Nucleotide Archive, with accession number PRJEB9878

### 4.3. Herbarium samples

Historic herbarium samples were either directly sampled by us in different herbaria both in North America and Europe, or sampled there by collection curators and sent to us by post (electronic supplementary material, table S1). The amount of tissue used for destructive sampling ranged from 2 to 8 mm^2^.

Modern herbarium samples were derived from a recent collection of *A. thaliana* wild populations in North America by the Max Planck Institute for Developmental Biology. After collection plant tissue was dried by pressing between acid-free papers using a wooden press for 6 weeks and subsequently mounted in herbarium sheets.

### 4.4. DNA extraction, library preparation and sequencing

#### 4.4.1. DNA extraction from historic herbarium samples

DNA extractions were carried out in clean room facilities in all cases. The majority of the samples were extracted following the PTB extraction protocol [16] as previously described [15]. Samples from the Cornell Bailey Hortorium were extracted using the CTAB extraction protocol [16] (electronic supplementary material, table S2).

#### 4.4.2. DNA extraction of modern herbarium samples

DNA extractions were carried out following the PTB extraction protocol [16].

#### 4.4.3. Library preparation historic samples

Illumina double indexed sequencing libraries [34,35] were prepared from each sample as previously described [15]. The excess of C-to-T substitutions associated with DNA damage and caused by deamination of cytosines [36] was not repaired in order to quantify the amount of damage present in samples of different ages.

#### 4.4.4. Library preparation modern herbarium samples

Indexed libraries were prepared using the Illumina TruSeq Nano DNA sample preparation kit following the manufacturer's instructions.

#### 4.4.5. Sequencing

Libraries were paired-end sequenced on the Illumina HiSeq 2000, HiSeq 2500 or MiSeq instruments (electronic supplementary material, table S2).

### 4.5. Read processing and mapping

#### 4.5.1. Historic herbarium samples

Reads were assigned to each sample based on their indices. Adapters were trimmed using the program Skewer (v. 0.1.120) with default settings with the natively implemented Illumina TruSeq adapter sequences [37]. Forward and reverse reads were merged using the program Flash (v. 1.2.11) with default settings, except for an elevated maximum overlap (100–150 bp depending on read length) to allow a more accurate scoring of highly overlapping read pairs [38]. Merged reads were mapped as single-end reads to their respective reference genomes: *Arabidopsis thaliana* [39,40], *S. tuberosum* [41], *S. lycopersicum* [42], *P. infestans* [43]. The mapping was performed using BWA-MEM (v. 0.7.10) with default settings [44]. PCR-duplicates were identified after mapping based on start and end coordinates and for every cluster of duplicate reads a consensus sequence was generated [45].

#### 4.5.2. Modern herbarium samples

Reads were processed very similarly to the reads that belong to historic samples. The vast majority of reads could not be merged, which indicates that the DNA was not as fragmented as in older herbarium samples. Therefore, we mapped the paired-end reads using BWA-MEM (v. 0.7.10) with default parameters [44] and inferred fragment size based on paired-end mapping.

### 4.6. Analysis of DNA damage patterns

#### 4.6.1. Fragment length

We analysed the fragment length distributions of merged reads. We fitted a lognormal distribution to the empirical fragment length distributions using the fitdistr function from the package MASS using R. Since in a lognormal distribution the logarithm of a variable is normally distributed, we used the mean of this distribution (log-mean) to summarize the fragment length distribution. The regression on the relationship among log-mean of fragment lengths and collection year was carried out using the lm function in R. For visualization (figure 1*b*), we used the fragment length median on a log-scaled *y*-axis, since the median is more intuitive to understand than the log-mean value. The relationship between log-mean and median follows the formula: median = e^log‐mean^.

#### 4.6.2. DNA break points

To analyse the nucleotide genomic context around DNA break points, we used the software mapDamage 2.0 (v. 2.0.2–12) [46]. mapDamage calculates the genomic base frequencies around mapped reads and within reads, which allows the inference of the bases most likely to be present before DNA break points. We calculated the relative enrichment of either adenine or guanine at the 5′-end (position −1 compared with position −5). The frequencies of both adenine and guanine were extracted from the output file dnacomp.txt produced by mapDamage. The regression on the relationship among purine relative enrichment (either adenine or guanine) and collection year was carried out using the lm function in R. The whole procedure was carried out for plant nuclear and chloroplast reads independently.

#### 4.6.3. Nucleotide misincorporation

All types of nucleotide misincorporations relative to the reference genome were calculated per library using mapDamage 2.0 (v. 2.0.2–12) [46]. The percentage of C to T substitutions at first base was extracted from the output file 5pCtoT_freq.txt produced by mapDamage. The regression on the relationship among the percentage of C to T substitutions at first base (5′-end) and collection year was carried out using the lm function in R. For the regression we used the percentage of deamination at first base. The whole procedure was carried out for plant nuclear and chloroplast reads independently, and also for pathogen nuclear reads in the case of samples infected with *P. infestans*.

### 4.7. Calculation of DNA decay rate

To calculate the decay rate of DNA retrieved from plant desiccated tissue, we used a previously described methodology [2] and adapted it to multiple samples. The random fragmentation of DNA molecules that occurs post-mortem follows a model of exponential decay, i.e. the amount amplifiable template decreases exponentially with increasing length [17]. We used the distributions of fragment length (*L*) of mapped reads to calculate the DNA decay rate, which is determined by the proportion of damage sites (*λ*). Thus, the process can be described using an exponential distribution:

4.1 $$F(L)=F_{0}\times e^{-\lambda L},$$

where *L* is the fragment length, *F*(*L*) the frequency of fragment with length *L* and *F*_0_ the frequency intersect at length 0.

After logarithmic transformation there is a linear relationship between the logarithms of the fragment frequency and fragment length with a slope −*λ*:

4.2 $$\log(F(L))=\log(F_{0})-\lambda L.$$

In this relationship, *λ* describes the fraction of bond survival per base in a single sample/library [2,17]. As previously described [2], the DNA decay rate per base per year, *k*, can then be calculated as:

4.3 $$k=\frac{\lambda }{\mathrm{age}}.$$

We calculated the decay rate across all analysed samples taking advantage of the negative correlation between fragment length and age of the sample. We plotted the damage fraction per site (*λ*) as a function of sample age. The slope of the linear regression on the relationship among *λ* and samples age yields *k*, the decay rate, according to the linear relationship:

4.4 λ=k×age.

The whole procedure was carried out for plant nuclear and chloroplast reads independently.

### 4.8. Analysis of covariance

To test if the regressions between chloroplast- and nuclear-derived reads were significantly different, we performed an analysis of covariance. We used the ‘aov’ function in R to test models where the sample age was the covariate and the DNA origin (chloroplast- or nuclear-derived) was the factor. In the first step, a model of type ‘*y* ∼ covariate × factor’ was used to include a possible interaction between covariate and factor, which would mean that there is a difference in the slope of the regression depending on the factor. If no significant interaction was detected, the ‘anova’ command in R was used to test this model against a model of type ‘*y* ∼ covariate + factor’. This last model does not include the interaction, therefore we can test whether the removal of the interaction has an effect on the fit of the model. If not, the second model was accepted with the conclusion that the regressions do not differ in slope, but possibly in their intersects (if there is a significant effect of the factor on the dependent variable *y*).

To test whether the linear regressions of read lengths and collection year between samples extracted with CTAB and PTB methods were different, we used the same approach as for chloroplast- and nuclear-derived reads. In this comparison, we used extraction method as the factor in the linear model.

## Supplementary Material

Figure S1: DNA fragmentation in a recently prepared herbarium sample Figure S2: DNA fragmentation of A. thaliana samples extracted using CTAB and PTB method Figure S3: DNA breaking points Figure S4: DNA break points and nucleotide misincorporation in a modern herbarium sample Figure S5: DNA breaking points in nuclear and chloroplast reads Figure S6: Nucleotide misincorporation in samples with lesions compatible with Phytophthora infestans Figure S7: . DNA decay and misincorporation in nuclear and chloroplast reads Table S1: Provenance of herbaria samples Table S2: Sequencing strategy and summary statistics

## Data accessibility

New DNA sequences are deposited in the European Nucleotide Archive, with accession no. PRJEB9878.
