# Supplementary material for: Temporal patterns of damage and decay kinetics of DNA retrieved from plant herbarium specimens
Source: R Soc Open Sci. 2016 Jun 22;3(6):160239. doi: 10.1098/rsos.160239 (PMC4929915; doi:10.1098/rsos.160239)
Supplement: Figure S1: DNA fragmentation in a recently prepared herbarium sample Figure S2: DNA fragmentation of A. thaliana samples extracted using CTAB and PTB method Figure S3: DNA breaking points Figure S4: DNA break points and nucleotide misincorporation in a modern herbarium sample Figure S5: DNA breaking [file rsos160239supp1.pdf]

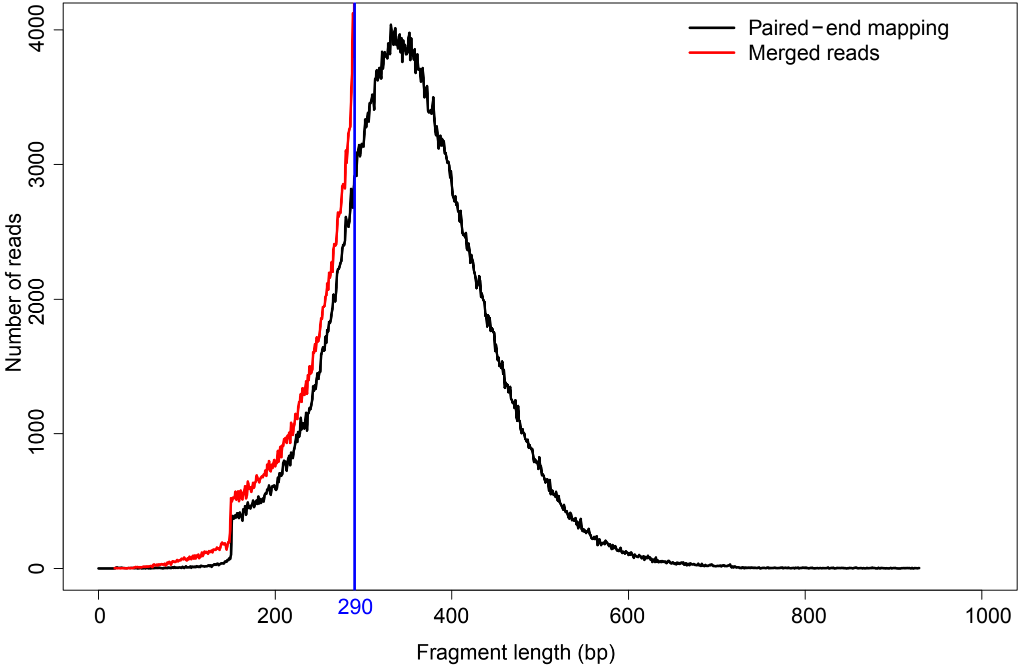
Paired−end mapping
Merged reads
4000
3000
2000
1000
0
Number of reads
0
200
290
400
600
800
1000
Fragment length (bp)

Fig. S2

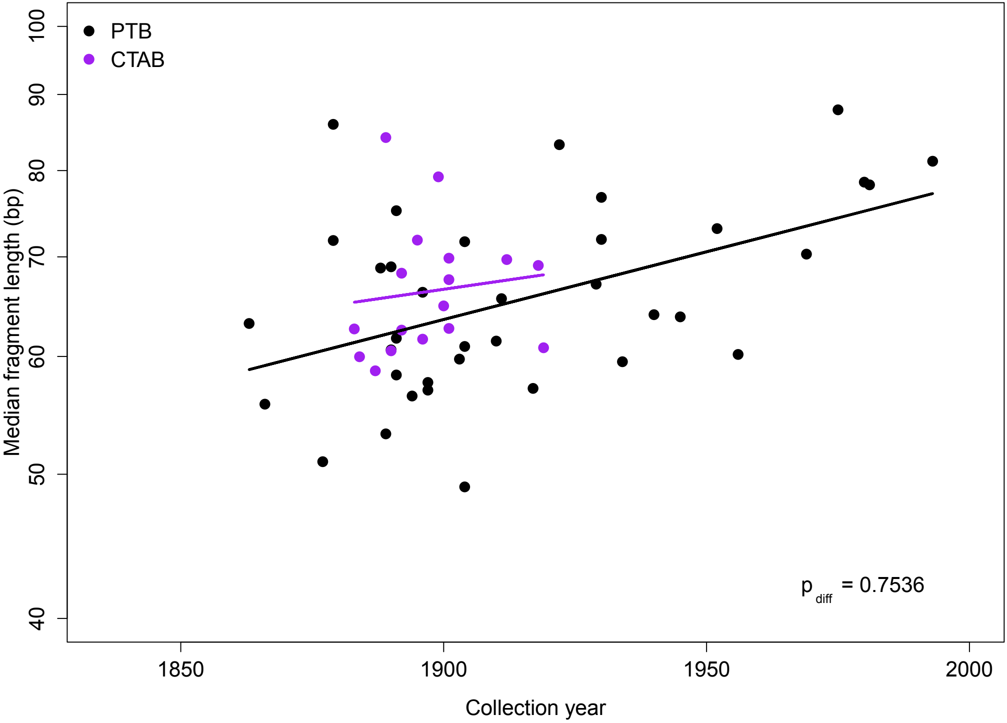

PTB
CTAB
100
90
80
70
60
50
40
Median fragment length (bp)
1850
1900
1950
2000
Collection year
p_{diff} = 0.7536

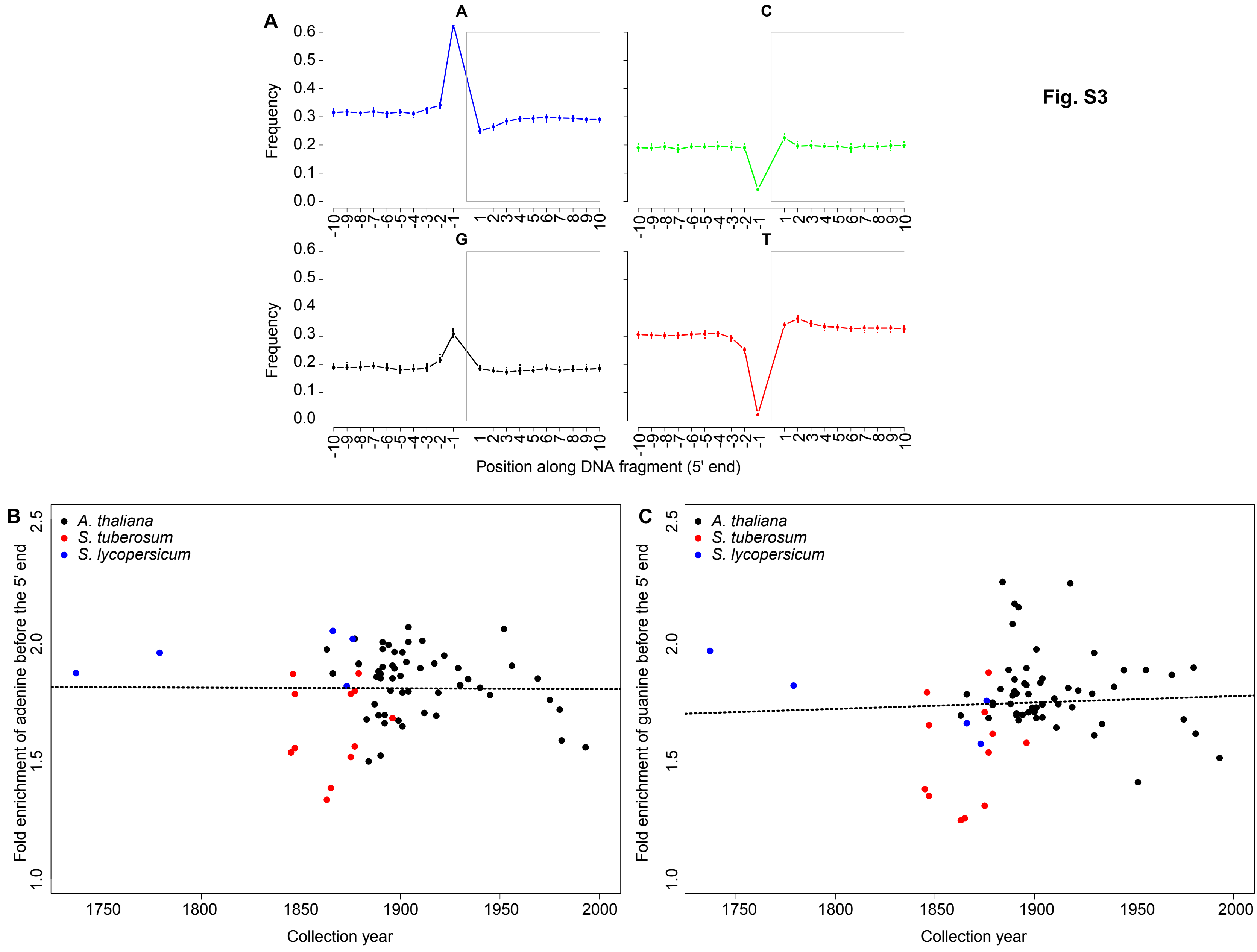

A
A
C
G
T
Frequency
Position along DNA fragment (5' end)
B
A. thaliana
S. tuberosum
S. lycopersicum
Fold enrichment of adenine before the 5' end
Collection year
C
Fold enrichment of guanine before the 5' end
Fig. S3

Fig. S4

A.

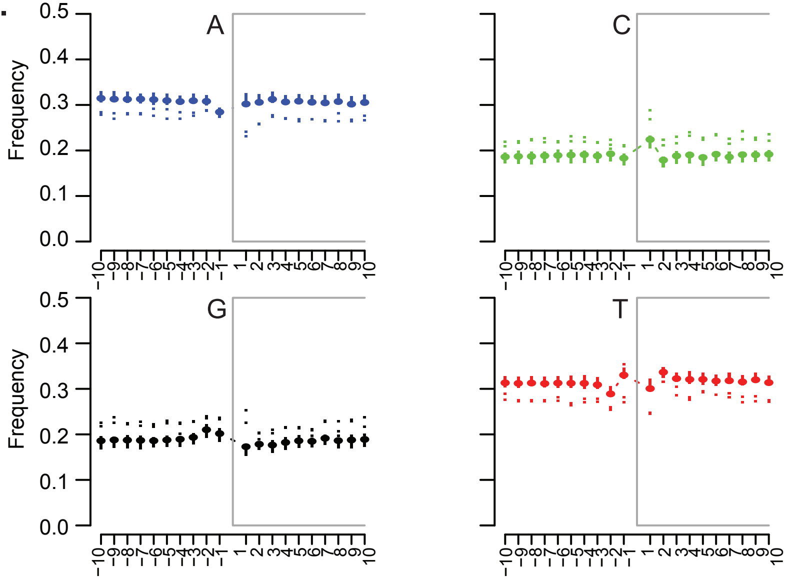

A
C
G
T
Frequency
0.0
0.1
0.2
0.3
0.4
0.5
−10 −9 −8 −7 −6 −5 −4 −3 −2 −1 1 2 3 4 5 6 7 8 9 10

B.

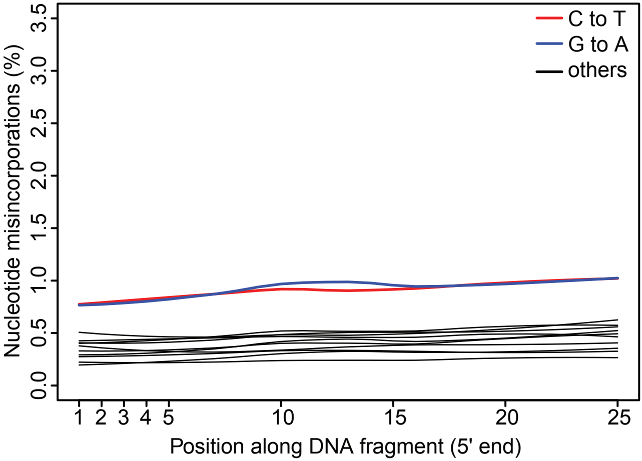

C to T
G to A
others
Nucleotide misincorporations (%)
0.0
0.5
1.0
1.5
2.0
2.5
3.0
3.5
1 2 3 4 5 10 15 20 25
Position along DNA fragment (5' end)

Fig. S5

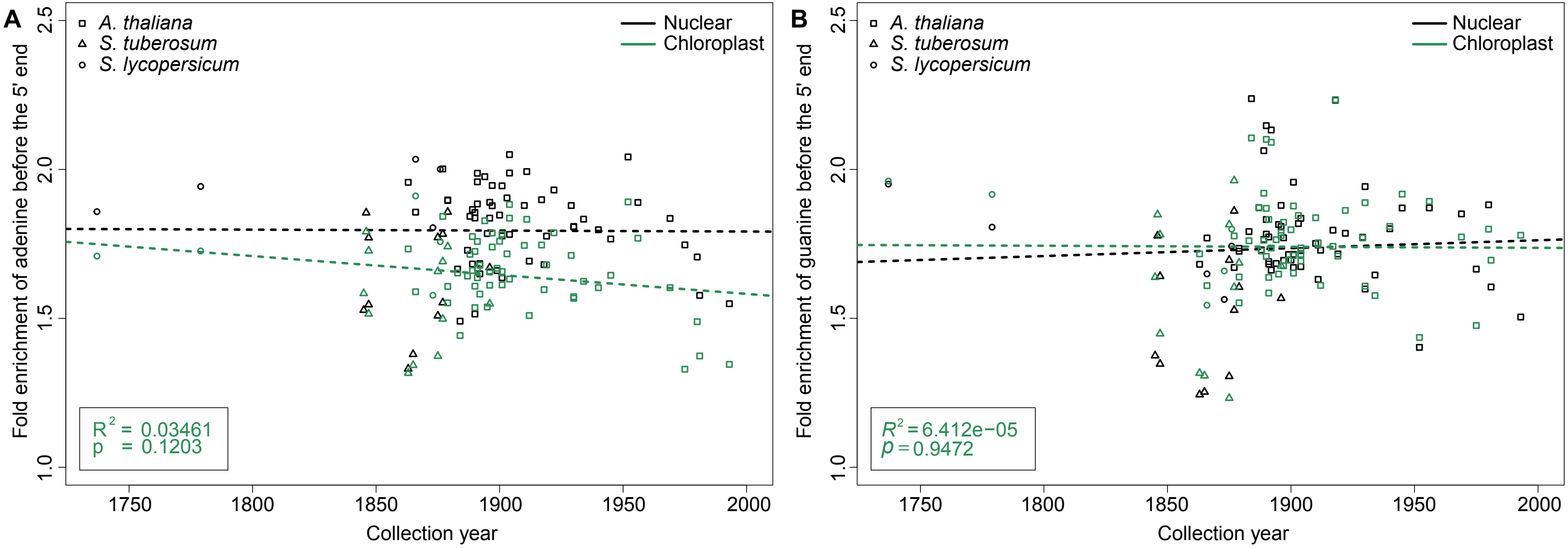
A
Fold enrichment of adenine before the 5' end
Collection year
A. thaliana
S. tuberosum
S. lycopersicum
Nuclear
Chloroplast
R2 = 0.03461
p = 0.1203
1.0
1.5
2.0
2.5
1750
1800
1850
1900
1950
2000
B
Fold enrichment of guanine before the 5' end
Collection year
A. thaliana
S. tuberosum
S. lycopersicum
Nuclear
Chloroplast
R2 = 6.412e−05
p = 0.9472

Fig. S6

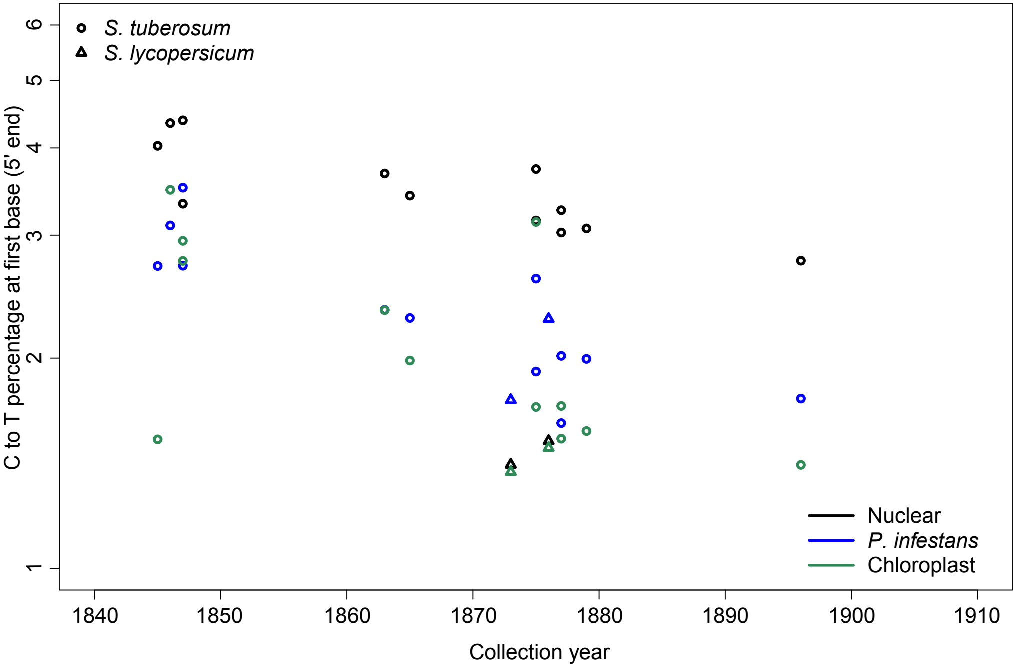
S. tuberosum
S. lycopersicum
Nuclear
P. infestans
Chloroplast
C to T percentage at first base (5' end)
1
2
3
4
5
6
1840
1850
1860
1870
1880
1890
1900
1910
Collection year

Fig. S7

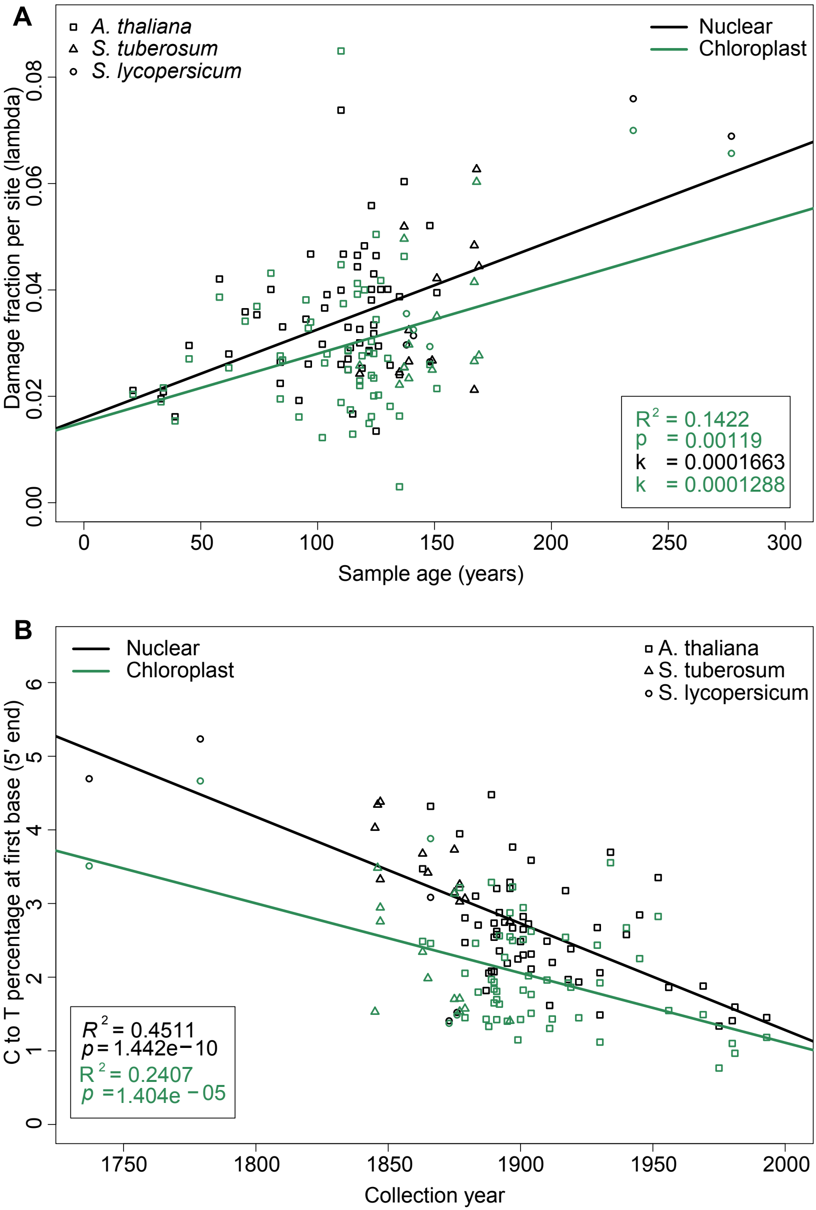
A
A. thaliana
S. tuberosum
S. lycopersicum
Nuclear
Chloroplast
Damage fraction per site (lambda)
0.00
0.02
0.04
0.06
0.08
0
50
100
150
200
250
300
Sample age (years)
R 2 = 0.1422
p = 0.00119
k = 0.0001663
k = 0.0001288
B
Nuclear
Chloroplast
A. thaliana
S. tuberosum
S. lycopersicum
C to T percentage at first base (5' end)
0
1
2
3
4
5
6
1750
1800
1850
1900
1950
2000
Collection year
R 2 = 0.4511
p = 1.442e−10
R 2 = 0.2407
p = 1.404e −05

Table S1. Provenance of herbaria samples.

ID	Country of origin	Collection year	Species	Reference
BH0000052681	USA	1919	*A. thaliana*	1
BH0000061407	USA	1890	*A. thaliana*	1
BH0000061409	USA	1892	*A. thaliana*	1
BH0000061416	USA	1892	*A. thaliana*	1
BH0000061457	USA	1900	*A. thaliana*	1
BH0000061454	USA	1884	*A. thaliana*	1
BH0000061397	USA	1899	*A. thaliana*	1
BH0000061417	USA	1912	*A. thaliana*	1
BH0000061449	USA	1901	*A. thaliana*	1
BH0000061420	USA	1896	*A. thaliana*	1
BH0000061395	USA	1883	*A. thaliana*	1
BH0000061424	USA	1895	*A. thaliana*	1
BH0000061393	USA	1918	*A. thaliana*	1
BH0000061399	USA	1901	*A. thaliana*	1
BH0000061448	USA	1901	*A. thaliana*	1
BH0000061418	USA	1887	*A. thaliana*	1
BH0000061406	USA	1889	*A. thaliana*	1
OSU46488	USA	1917	*A. thaliana*	2
OSU13896	USA	1930	*A. thaliana*	2
OSU13900	USA	1934	*A. thaliana*	2
OSU54623	USA	1956	*A. thaliana*	2
OSU79944	USA	1911	*A. thaliana*	2
OSU183663	USA	1969	*A. thaliana*	2

OSU361885	USA	1930	*A. thaliana*	2
OSU150287	USA	1980	*A. thaliana*	2
OSU163761	USA	1981	*A. thaliana*	2
OSU364632	USA	1993	*A. thaliana*	2
UNC54051	USA	1945	*A. thaliana*	3
UNC25707	USA	1940	*A. thaliana*	3
UNC63978	USA	1910	*A. thaliana*	3
CT79391	USA	1904	*A. thaliana*	4
CT79409	USA	1929	*A. thaliana*	4
CT79389	USA	1975	*A. thaliana*	4
176849CFM	USA	1904	*A. thaliana*	5
531679CFM	USA	1922	*A. thaliana*	5
1507461CFM	USA	1952	*A. thaliana*	5
NY102365	USA	1903	*A. thaliana*	6
NY1365344	USA	1890	*A. thaliana*	6
NY888124	USA	1863	*A. thaliana*	6
NY1365363	USA	1888	*A. thaliana*	6
NY888144	USA	1866	*A. thaliana*	6
NY1365364	USA	1889	*A. thaliana*	6
NY888134	USA	1877	*A. thaliana*	6
NY1365332	USA	1890	*A. thaliana*	6
NY1365337	USA	1891	*A. thaliana*	6
NY1365370	USA	1897	*A. thaliana*	6
NY1365333	USA	1894	*A. thaliana*	6
NY1365374	USA	1896	*A. thaliana*	6
NY102364	USA	1904	*A. thaliana*	6
NY1365375	USA	1897	*A. thaliana*	6

NY888141	USA	1879	*A. thaliana*	6
NY1365354	USA	1891	*A. thaliana*	6
NY888141	USA	1879	*A. thaliana*	6
NY1365354	USA	1891	*A. thaliana*	6
KM177509	England	1865	*S. tuberosum*	7
KM177517	Wales	1875	*S. tuberosum*	7
KM177514	Ireland	1847	*S. tuberosum*	7
KM177500	England	1845	*S. tuberosum*	7
KM177513	Ireland	1846	*S. tuberosum*	7
KM177548	England	1847	*S. tuberosum*	7
M-0182898	Germany	1863	*S. tuberosum*	8
M-0182906	Germany	1877	*S. tuberosum*	8
M-0182907	Germany	1875	*S. tuberosum*	8
M-0182896	Germany	1877	*S. tuberosum*	8
M-0182903	Canada	1896	*S. tuberosum*	8
M-0182904	Austria	1879	*S. tuberosum*	8
BM000777791	UK	1866	*S. lycopersicum*	9
BM000815937	UK	1737	*S. lycopersicum*	9
BM000849510	UK	1779	*S. lycopersicum*	9
M-0182897	USA	1876	*S. lycopersicum*	8
M-0182900	Germany	1873	*S. lycopersicum*	8
Modern herbarium				
INTHRR_108	USA	2014	*A. thaliana*	10
INTHRR_110	USA	2014	*A. thaliana*	10
INTHRR_54	USA	2014	*A. thaliana*	10
MAAA_16	USA	2014	*A. thaliana*	10
MAAA_41	USA	2014	*A. thaliana*	10

MAAA_61	USA	2014	*A. thaliana*	10
MDTCR_32	USA	2014	*A. thaliana*	10
MDTCR_42	USA	2014	*A. thaliana*	10
MDTCR_51	USA	2014	*A. thaliana*	10
NCARS_32	USA	2014	*A. thaliana*	10
NCARS_51	USA	2014	*A. thaliana*	10
NCARS_9	USA	2014	*A. thaliana*	10
NYBG_154	USA	2014	*A. thaliana*	10
NYBG_157	USA	2014	*A. thaliana*	10
NYBG_169	USA	2014	*A. thaliana*	10

[*]1. Cornell Bailey Hortorium; 2.Ohio State University Herbarium; 3. University of North Carolina Herbarium; 4. University of Connecticut Herbarium; 5. Chicago Field Museum; 6. New York Botanical Garden; 7. Kew Royal Botanical Garden; 8. Botanische Staatssammlung München; 9. Natural History Museum, London; 10. Max Planck Institute for Developmental Biology collection.

Table S2. Sequencing strategy and summary statistics.

ID	Read type	Number raw read pairs	Percentage merged reads	Percentage of reads mapped to reference
BH0000052681	2x101	4,070,122	99.7	53.5
BH0000061407	2x101	4,083,714	99.7	28.4
BH0000061409	2x101	4,316,387	99.5	66.5
BH0000061416	2x101	3,927,269	99.5	71.3
BH0000061457	2x101	478,438	99.4	74.6
BH0000061454	2x101	497,373	99.4	66.6
BH0000061397	2x101	589,715	99.3	89.5
BH0000061417	2x101	3,310,517	99.3	76.9
BH0000061449	2x101	4,873,199	98.9	92.4
BH0000061420	2x101	4,137,492	98.8	81.0
BH0000061395	2x101	608,442	98.8	87.6
BH0000061424	2x101	560,391	98.8	83.4
BH0000061393	2x101	583,208	98.7	81.7
BH0000061399	2x101	5,030,578	98.4	88.5
BH0000061448	2x101	3,250,580	98.2	60.9
BH0000061418	2x101	4,110,260	98.2	72.3
BH0000061406	2x101	5,525,374	98.0	85.2
OSU46488	2x150	775,962	97.9	69.3
OSU13896	2x150	1,222,514	97.9	73.5
OSU13900	2x150	885,259	97.8	42.0
OSU54623	2x150	814,706	97.7	69.1
OSU79944	2x150	935,769	97.7	76.5

OSU183663	2x150	941,643	97.5	68.6
OSU361885	2x150	815,261	97.5	74.9
OSU150287	2x150	1,100,356	97.3	68.8
OSU163761	2x150	1,026,456	97.1	85.0
OSU364632	2x150	992,421	97.1	68.7
UNC54051	2x150	998,331	96.9	86.7
UNC25707	2x150	741,049	96.7	71.6
UNC63978	2x150	987,305	96.7	74.4
CT79391	2x150	928,321	96.5	70.7
CT79409	2x150	854,432	96.4	81.8
CT79389	2x150	781,485	96.3	30.4
176849CFM	2x150	918,792	96.2	61.5
531679CFM	2x150	870,119	96.2	83.2
1507461CFM	2x150	924,012	96.2	78.4
NY102365	2x150	1,313,215	96.2	67.9
NY1365344	2x150	1,496,570	96.0	80.6
NY888124	2x150	2,083,306	95.9	70.0
NY1365363	2x150	1,408,827	95.7	74.9
NY888144	2x150	1,664,389	95.7	64.6
NY1365364	2x150	1,660,257	95.7	60.3
NY888134	2x150	1,333,231	95.6	75.9
NY1365332	2x150	2,093,699	95.5	80.5
NY1365337	2x150	1,773,246	95.5	81.3
NY1365370	2x150	1,322,896	95.5	80.1
NY1365333	2x150	1,937,923	95.2	79.4
NY1365374	2x150	1,785,933	95.0	75.9
NY102364	2x150	1,621,005	95.0	59.2

NY1365375	2x150	1,713,640	94.8	36.3
NY888141	2x101	12,574,596	94.8	83.9
NY1365354	2x101	10,832,427	94.8	66.5
NY888141	2x101	9,605,582	94.7	86.1
NY1365354	2x101	11,982,636	94.5	78.9
KM177509	2x151	666,206	94.5	70.1
KM177517	2x151	1,017,778	94.4	62.6
KM177514	2x151	803,024	94.4	53.5
KM177500	2x151	808,056	94.4	30.8
KM177513	2x151	704,372	94.3	60.0
KM177548	2x151	611,321	94.0	53.9
M-0182898	2x101	2,206,547	93.9	65.0
M-0182906	2x101	2,441,550	93.7	75.6
M-0182907	2x101	2,224,182	93.2	75.4
M-0182896	2x101	2,735,620	93.1	55.1
M-0182903	2x101	3,668,118	92.9	65.3
M-0182904	2x101	2,989,144	92.5	83.7
BM000777791	2x101	533,872	91.9	91.9
BM000815937	2x101	554,635	91.6	66.1
BM000849510	2x101	519,254	87.1	68.9
M-0182897	2x101	3,775,244	85.1	79.7
M-0182900	2x101	1,860,985	83.5	85.7
Modern Herbarium				
INTHRR_108	2x150	1,013,210	17.1	85.5
INTHRR_110	2x150	614,641	17.5	76.6
INTHRR_54	2x150	1,035,098	17.7	80.8
MAAA_16	2x150	816,998	18.5	90.3

MAAA_41	2x150	994,861	19.8	68.0
MAAA_61	2x150	1,029,669	18.7	90.0
MDTCR_32	2x150	805,969	15.8	87.0
MDTCR_42	2x150	977,367	21.1	91.5
MDTCR_51	2x150	175,376	20.4	90.7
NCARS_32	2x150	748,794	18.9	86.8
NCARS_51	2x150	949,138	22.8	88.8
NCARS_9	2x150	787,902	17.7	85.0
NYBG_154	2x150	1,867,301	25.9	68.1
NYBG_157	2x150	2,001,726	40.0	62.0
NYBG_169	2x150	1,691,752	29.8	75.8
